# The strategic trajectory of artificial intelligence in Qatar’s healthcare sector: a model for UN Sustainable Development Goal 9

**DOI:** 10.3389/frai.2026.1702242

**Published:** 2026-01-27

**Authors:** Mohamed A. Ismail

**Affiliations:** Hamad Medical Corporation, Doha, Qatar

**Keywords:** artificial intelligence, digital transformation (DT), healthcare, Qatar, SDG 9

## Abstract

This narrative review is first of its kind to strategically position AI and healthcare in the Qatari state under the broad framework of QNV-2030. The review highlights Qatar’s forward-looking digital infrastructure upon which its AI initiatives are being built. The paper lists several innovative clinical process developments such as precision medicine and AI-based diagnostics. Furthermore, it analyzes the country’s strong ethical and regulatory frameworks underpinning AI adoption, including aspects related to privacy and security. While these aspects are positive, the review identifies areas of concern wherein improvements may be effected: a deeper critical analysis of implementation challenges, greater integration of empirical evidence, and strategies for human capital development beyond mere upskilling. This paper serves as an exemplary case study, demonstrating how a nation can strategically align its top-down initiatives, digital infrastructure investments, and forward-looking regulatory environment with the United Nations Sustainable Development Goal 9 (SDG 9), which focuses on Industry, Innovation, and Infrastructure.

## 1 Introduction

**Figure 1:**
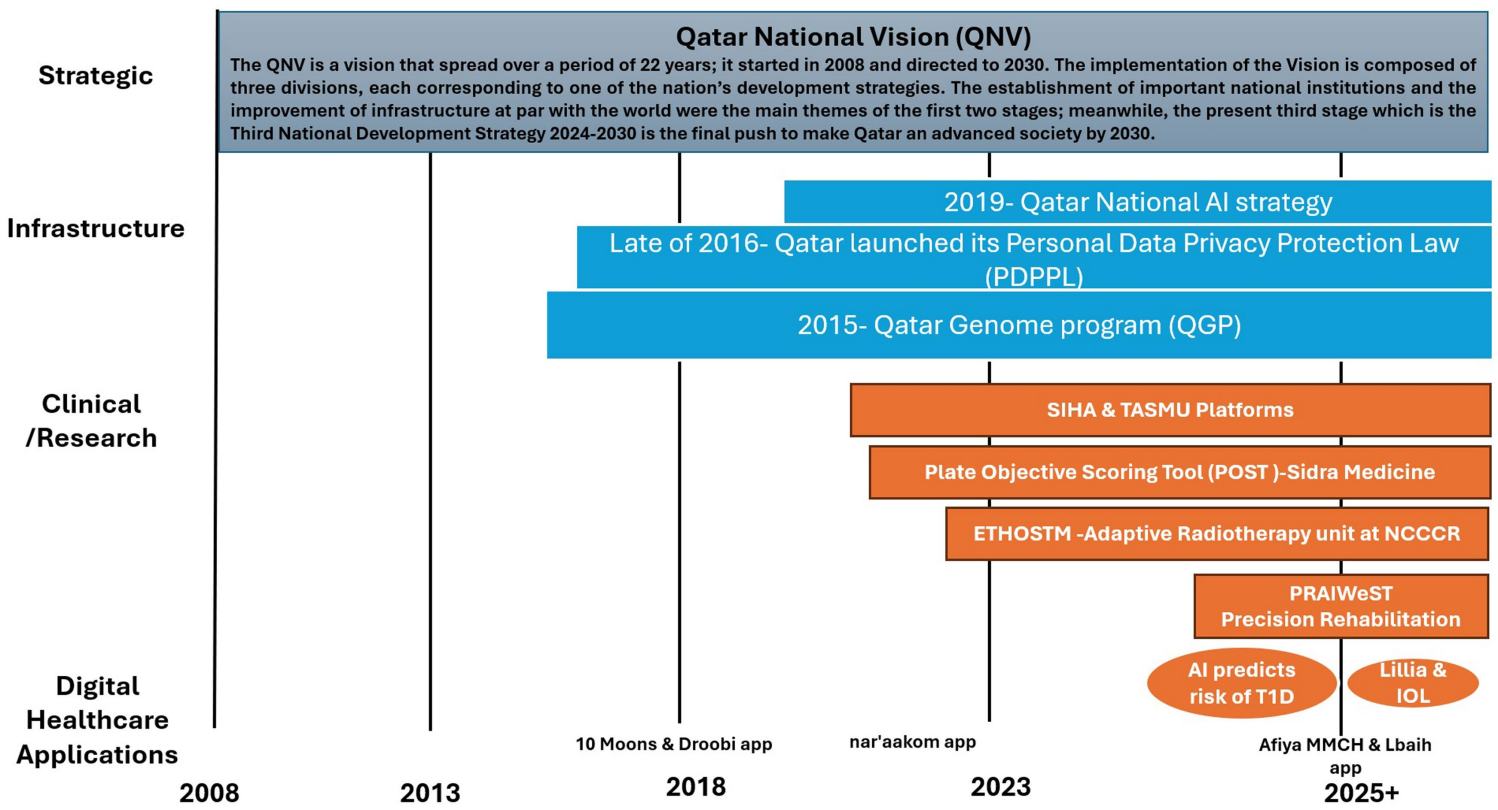
Timeline of major AI applications within healthcare initiatives in Qatar.

Qatar’s health sector is a highly developed and rapidly evolving system, characterized by a dual structure comprising robust public and private sectors (Goodman, 2015). Beyond data analysis, AI extends to robotics for surgical procedures and hospital logistics (Cruz et al., 2024). These AI systems improve patient outcomes by assisting in the correct diagnosis of patients or by ensuring ease of care delivery (Alowais et al., 2023). Human development is central to all healthcare and AI development ventures. This vision entails a highly skilled and innovative population that translates the national vision into tangible goals through a series of subsequent national development plans, upon which the recently launched National Health Strategy (2024–2030) and National Development Strategy (NDS3) 2024–2030 are built (MOPH, 2025). This review shows that Qatar’s proactive investments in its digital infrastructure, such as electronic health record systems (EHR) and high-speed networks directly align with SDG 9’s call for strong, reliable infrastructure. Furthermore, this paper’s findings on the Qatar Genome Project (QGP) and the use of AI for precision medicine demonstrate how Qatar is fostering innovation to improve health outcomes. This also shows a form of modern, sustainable “industrialization” in the healthcare sector, moving it toward a more efficient, technology-driven model. Figure 1 provides a chronological overview of these foundational milestones, highlighting the latest AI programs.

This review will briefly discuss the strategic imperative, current initiatives, the dilemma of regulatory and ethical landscape ending with opportunities, challenges, and strategic recommendations. To address the current stage of AI adoption, all identified initiatives were categorized into two distinct classes during synthesis: (A) Strategic Commitments, supported by government-issued mandates (the ‘intent’); and (B) Empirical Successes, substantiated by available published, peer-reviewed data demonstrating validated outcomes or documented pilot results (the ‘impact’). The detailed methodology, including comprehensive literature search, specific databases used, and full inclusion and exclusion criteria for this policy-oriented narrative synthesis, are provided in Supplementary file 1.

## 2 The strategic imperative: Qatar’s vision for a future-ready healthcare system

Qatar’s milestones in healthcare AI aren’t fueled by a single recent push, but rather through national development strategies, where the vision is broken down into actionable goals for the intermediate term (each stage with a five-year plan) (MOPH, 2018).

### 2.1 The national AI and digital agenda: enabling transformation

The 2019 National AI Strategy, devised as an extension of QNV 2030 to ensure economic diversification and increase technological competence, provides a roadmap of AI adoption. The document is built on six strategic pillars: Education and talent development, data access, employment transformation, new business and economic opportunities, a focus on key sectors, and ethics and public policy (MCIT, 2025a). Furthermore, in 2021 the Artificial Intelligence Committee was established to oversee the integration of AI across all sectors in the country (Qatar M., 2025). By emphasizing the strategy of AI in all aspects of human activities, “AI+X,” the country demonstrates its strong willingness to achieve it. Precision medicine is a strategic focus, showcasing a structured approach to AI in healthcare. This is supported by the Digital Agenda 2030 that also supports the use of AI in the digital transformation of government, healthcare, and finance (Aboulnaga et al., 2021).

### 2.2 Governance and coordination: a centralized engine

To illustrate this strategic alignment, the following Table 1 shows how high-level visions correspond with specific, actionable strategies and initiatives.

**Table 1:** Strategic alignment of Qatar’s national vision and health strategies with key pillars of healthcare AI implementation.

Strategic level	Strategy	Key pillars	Link to healthcare AI
National vision	Qatar national vision 2030	Human development	A core goal is to cultivate a highly skilled population and ensure high-quality healthcare for all residents.
Sectoral strategy	National Health Strategy (NHS) 2018–2022	Integrated Model of high-quality care	Optimizing information systems, telemedicine, and digital health solutions to deliver an integrated, patient-centered model of care.
	National Health Strategy (NHS) 2024–2030	Improved population health and wellbeing Excellence in service delivery and patient care Health system efficiency and resilience	AI is integrated to support strategic themes, including enhancing quality and accessibility of care, driving clinical excellence, and improving the sustainability of the integrated healthcare model through innovation and digital solutions.
Technological roadmap	National AI strategy 2019	Focus areas for Qatar	Precision medicine is identified as a key strategic area for AI development and adoption.
Operational governance	AI committee 2021	Implement and oversee AI strategy	The committee is tasked with coordinating AI initiatives and ensuring their execution across sectors like healthcare.

This table illustrates the top-down strategic framework, showing how high-level national goals and sectoral health strategies are translated into actionable pillars that support the development and adoption of artificial intelligence (AI) in the healthcare sector.

### 2.3 The role of the Qatar Foundation and QRDI council in catalyzing AI/ML

The Qatar Foundation (QF) acts as the primary institutional catalyst for the nation’s research and innovation ecosystem (QF, 2025). QFs commitment is executed through its dedicated research arm, the Qatar Research, Development and Innovation (QRDI) Council (formerly the Qatar National Research Fund, QNRF), which aligns its funding priorities directly with the national strategies, including the Qatar National Vision 2030 and the National AI Strategy (QRDI, 2025). The QRDI Council’s funding mechanisms are explicitly designed to foster a knowledge-based economy by supporting investigator-led research, promoting collaboration between academia and industry, and addressing key national challenges in sectors such as health, digital technology, and sustainability (QRDI, 2025). One of these projects is the startup Lillia (formerly Droobi). Lillia’s AI-powered platform leverages ML algorithms, paired with human expert coaching, to manage chronic conditions like diabetes, demonstrating a shift toward a proactive and personalized care model. The development of a chronic care digital twin represents a significant step in applying advanced AI for real-time risk monitoring and precision medication, directly supporting the manuscript’s theme of strategic AI adoption in healthcare (GM, 2025).

## 3 Building the foundation: digital transformation and infrastructure

### 3.1 The digital bedrock: EHR and beyond

Qatar has established a national EHR system that covers a remarkable percentage of healthcare institutions in the country and is considered as prime example of building the “resilient infrastructure” called for by SDG 9 (HMC, 2017; SDGs, 2025). It allows the real-time retrieval and cross-verification of data among multiple providers. It serves as a critical data source for creating meaningful AI models for medicine, particularly in life critical applications. While the national EHR system is a landmark achievement, its utility for AI model development is not without challenges. Specifically, the heterogeneity of data entry, issues of interoperability across all private and public entities, and the need for extensive data cleaning and standardization represent significant, ongoing implementation hurdles that must be overcome to fully realize the potential of AI in life-critical applications.

### 3.2 A comprehensive healthcare ecosystem ripe for AI integration

Qatar’s healthcare system is characterized by a comprehensive, dual-sector infrastructure encompassing both public and private networks. This vast ecosystem, with innumerable hospitals, clinics, and health centers, provides probably the largest framework on which AI technologies can be deployed and scaled. While the public sector was actively, maintaining 19 hospitals and 35 public health centers, the private sector is also incubating at a faster rate; hence, AI-based solutions can integrate to cover a wide patient base across a varied spectrum of care settings (QNA, 2025). A strong digital foundation has been positioned with the National EHR system and a complete healthcare ecosystem, which serves as the essential launchpad for Qatar’s cutting-edge AI projects. The following section details how this foundation is being leveraged to drive frontier innovation across clinical practice and research, from precision medicine to advanced diagnostics.

## 4 Frontier innovation: AI in clinical practice and research

### 4.1 The cornerstone of precision health: the Qatar Genome Program

The QGP marks the initial step toward implementing AI-driven precision medicine and is a direct reflection of SDG 9’s focus on fostering innovation and enhancing scientific research (Fakhro, 2016). The QGP, a national project, aims to build genomic databases of the Qatari people and the Arab population for researchers. This initiative tackles the important ethical and technical issue of algorithmic bias in AI-driven healthcare. AI models that are developed on datasets of a particular race, for instance, a European race, work poorly on a different ethnic group. The QGP is designed to build datasets for the local and regional populations, ensuring that the models developed are not only functionally better but also equitable and relevant to the people they intend to serve thereby aligning with the ethical principle of ‘Justice’ and helping to prevent the increase in healthcare inequalities (Mbarek et al., 2022).

### 4.2 Breakthroughs in diagnostics and predictive health

Qatar’s leading research institutions are leveraging AI to address complex health issues, focusing on three key areas: AI in Diagnosis, AI Models for Disease Prediction and Treatment and AI Healthcare Applications.

#### 4.2.1 Cutting-edge applications: AI in precision medicine and diagnostics

Sidra Medicine and its partners have developed an AI tool designed for the early detection of Type 1 Diabetes (T1D) stands as the most extraordinary and innovative achievement of Sidra Medicine and its international collaborators. The tool, which evaluates microRNAs to create a ‘dynamic risk score’ using an AI-driven algorithm, has shown promising predictive capabilities in a peer-reviewed publication. The model’s performance in T1D stratification was demonstrated in a multi-context validation dataset (*n* = 662). The model shows strong predictive performance, successfully classifying individuals at high and low risk for T1D with an 84% true positive rate. Significantly improving upon traditional static risk models. Furthermore, the model demonstrated predictive utility in a clinical setting, specifically in assessing the response to islet transplantation therapy (Joglekar, 2025). This illustrates AI’s real-world clinical impact by shifting care from a reactive to a proactive and preventive model.

In the field of diagnostic scoring and medical imaging, Sidra Medicine is utilizing AI to analyze complex imaging data for early diagnosis and personalized treatment plans utilizing 3D printing for surgical planning, which relies on AI to process scans (Medicine Sidra, 2025a, 2025b). Research conducted by clinicians at Sidra Medicine has successfully validated an AI-driven methodology designed to objectively quantify critical anatomical variables from pre-operative imaging. This innovation directly impacts surgical decision-making and enhances the prediction of patient outcomes, particularly in complex pediatric procedures such as hypospadias repair. The core technology supporting this advancement is the Plate Objective Scoring Tool (POST). This system employs a deep convolutional neural network (CNN) to automatically and reproducibly measure key phenotypic landmarks of the urethral plate from standard two-dimensional images (Abbas, 2021, 2022). While general literature supports the hypothesis that 3D printing in surgical planning leads to better outcomes, the validated studies at Sidra Medicine primarily focus on the AI-driven diagnostic (POST) and the establishment of the integrated EMR/3D printing workflow. This integrated approach positions the institution to systematically capture outcome data from 3D-planned surgeries for future empirical validation.

The National Center for Cancer Care and Research (NCCCR) at HMC is implementing advanced radiotherapy technology, equipped with AI, to serve cancer patients with personalized treatment plans. This particular AI system recalibrates and optimizes treatment every day to the patient’s updated anatomy to enable precise delivery of radiation to the tumor and better clinical outcomes. In addition, the Center of Clinical Precision Medicine and Genomics at HMC is integrating AI into its pharmacogenomics research and other workflows to further enhance individualized patient care (HMC, 2022, 2025a). NCCCR has successfully adopted the Varian Ethos system to implement online adaptive radiotherapy (OART), a pivotal advancement in precision oncology. This technology leverages AI for automated contouring and real time treatment plan adaptation, directly improving the efficiency and accuracy of daily radiation delivery. Empirical data published by NCCCR researchers validate the system’s dosimetric superiority (Gurusamy et al., 2024). Further research involving the integration of knowledge-based planning (RapidPlan) with the Ethos auto-planning workflow for both Head and Neck (H&N) and Prostate cancer confirmed its ability to optimize treatment quality. This integration resulted in a measurable reduction in the mean doses to OARs by an average of 9%, while meticulously maintaining highly consistent target coverage (Yoganathan et al., 2025).

The teams from Qatar University (QU) are also cooperating to address the significance of AI for other medical issues such as colorectal cancer. The research unit at QU is leveraging AI, mainly in the area of Drug Discovery and Repurposing, where algorithms quickly identify and create new therapeutic molecules or repurpose existing drugs. The process is highly optimized through Molecular Modeling, which uses AI for the difficult tasks of molecular docking and dynamic simulations. The simulations give an almost accurate prediction of the interaction between the potential drug candidates and the important pathways that are responsible for the cancer’s growth signaling, thus enabling an intelligent, targeted optimization of drug design. Moreover, AI is helping in the preclinical stage by providing support for the development and refinement of highly reliable rodent models that almost exactly resemble human CRC, which is crucial for the success of in-vivo testing. Lastly, AI is being utilized in Advanced Diagnostics, where its capacity to process large volumes of patient blood sample data is essential in identifying new, earlier-stage CRC biomarkers, thus promising to change the face of clinical screening (Crovella, 2025). Moreover, QU researchers are developing solutions with AI and semantic segmentation to enhance the assessment of embryo morphology for better infertility care and *in vitro* fertilization (IVF) success rates (Qatar University, 2024). The core objective of the study was to facilitate the shift toward a safer, more effective single viable blastocyst transfer by developing a reliable method for selecting the best embryo. The proposed solution involves leveraging AI and specifically Semantic Segmentation, a deep learning technique, to provide an automated, objective, and fast morphological analysis of the blastocyst’s internal components, such as the Inner Cell Mass (ICM), Trophectoderm (TE), and Zona Pellucida (ZP) (Qatar University, 2024). AI is poised to revolutionize neonatal care, particularly in Neonatal Intensive Care Units (NICUs), by dramatically improving the diagnosis, treatment, and early prevention of potential problems in newborns. Aligning with the global trend which Qatar is expected to embrace under initiatives like those referenced by TASMU SMART QATAR. Future efforts will focus on AI-driven solutions for the rapid detection of critical illnesses in at-risk infants, such as sepsis, respiratory distress, and neurological disorders (Qatar T. S., 2025).

#### 4.2.2 AI models for disease prediction

The QCRI has partnered with HMC in the creation of AI models that utilize a centralized EHR system to provide predictions about the onset and severity of various illnesses (HBKU, 2025b; HMC, 2025b). This model includes the prediction of cardiovascular events and the analysis of obesity through advanced multi-omics data, something that a healthcare specialist would not be able to accomplish on their own (El-Menyar et al., 2020). QCRI’s work also extends to a global context, as its scientists developed an AI model that successfully identified two drugs for COVID-19 treatment (HBKU, 2025b). This innovative use of AI in predictive healthcare extends beyond general medicine into specialized fields like maternal care. One notable use of AI in maternal care revolves around crafting predictive models for different maternal outcomes. Research is ongoing to determine how well AI models can forecast the method of delivery, be it vaginal or cesarean. When made, such predictions could significantly help clinicians as they prepare for births and navigate any arising challenges, provided the predictions are both accurate and comply with ethical standards (AlSaad et al., 2025). Furthermore, the integration of AI extends beyond diagnostics and genomics to encompass advanced therapeutic and rehabilitative applications, further solidifying Qatar’s strategic position as a hub for health technology innovation. Research from the QCRI at HBKU highlights the practical application of AI in clinical settings, including disease prediction using EHR and the automation of reporting in radiology to enhance diagnostic efficiency (Mir et al., 2022). Complementing this research, HMC has launched a pioneering Precision Rehabilitation Program that leverages state-of-the-art technologies, such as robotics, exoskeletons, and wearable devices, to tailor recovery plans for patients (HMC, 2024). This initiative is supported by academic literature, which recognizes AI’s critical role in rehabilitation for personalized care, outcome prediction, and real-time patient monitoring, demonstrating a mature transition of AI from the lab to the bedside in therapeutic and recovery workflows (Alshami et al., 2025).

#### 4.2.3 Qatar’s digital healthcare strategy (telemedicine growth/patient-centered digital tools)

Building strategically on this digital base, Qatar has invested further in upgrading patient care through digital solutions. Telemedicine in Qatar has grown significantly during the past years, with a massive shift to remote clinical care during the Covid pandemic. While records showed a very significant increase in teleconsultations during 2020, there has been a steady dependence on digital care model since then (AlAhmad et al., 2021). By mid-2025, HMC launched a mobile app ‘Lbaih’ as an example of this digital shift, with the unique ability for patients to oversee their care and instantly retrieve their data, the app elevates patients to the status of “active partners” in their healthcare (HMC, 2025c). Table 2 summarizes digital healthcare applications in Qatar.

**Table 2:** Key digital healthcare applications and initiatives in Qatar.

Initiative/program	Organization/source	Type of digital health	Key details and impact
10 Moons (Sidra, 2018).	Sidra Medicine	Patient-centric mHealth (maternity)	Qatar’s first in-house developed, bilingual maternity app. Tracks pregnancy, provides personalized guidance, and synchronizes with hospital admission systems. It highlights the use of digital technology to streamline clinical processes and improve patient.
PHCC and Droobi Health Partnership (PHCC, 2020).	PHCC	AI-enabled Digital Therapeutics	Collaboration to offer digital health programs for the proactive management of Chronic conditions like Type 2 Diabetes Mellitus. Exemplifies the integration of AI driven, Personalized care models into the public PHC system.
Nar’aakom (PHCC, 2020).	PHCC	Patient Portal	Provides a bilingual, centralized platform for patients to manage appointments, access health records, and connect with their assigned family physician. Supports universal health coverage and Improved accessibility at the primary care level.
Afiya MMCH (Times, 2025).	Military medical city hospital (MMCH)	Comprehensive Digital solution	Manages daily tasks like appointment scheduling, remote live medical consultations, accessing test results and medical records, and tracking treatment plans.
‘Lbaih’ Mobile App (HMC, 2025c).	Hamad medical corporation	Patient Portal/mobile health (mHealth)	Allow HMC patients to book consultations, view medical records, and fetch key healthcare data (vital statistics, lab results, prescribed medications).

This table details the current landscape of digital health solutions, identifying the implementing organization, the type of digital health focus (e.g., mHealth, Digital Therapeutics), and the specific impact of each program on patient care and clinical processes.

#### 4.2.4 AI healthcare applications

Beyond diagnosis and treatment, Qatari institutions are applying AI to a wider range of healthcare challenges. Furthermore, QCRI is developing AI-driven systems to improve public health, including a decision support system for clinicians called System for Integrated Health Analytics (SIHA) that uses data from wearable devices. Researchers are also focused on developing AI tools for the early detection of autism and creating assistive systems for people with visual impairments. The TASMU Smart Qatar program is exploring AI-assisted diagnosis through digital twin technology, where a virtual model of a patient is created to predict health risks and disease progression in real-time (HBKU, 2025c). Projects such as “AMAL-For-Qatar” establish the clear intent of the nation to step into the prenatal care revolution through advanced AI integration. The initiatives aim to harness AI-mediated approaches for precision medicine while improving monitoring and management of high-risk pregnancies.

#### 4.2.5 AI integration in Qatar’s private healthcare sector

While public institutions drive the foundational digital transformation, the private healthcare sector in Qatar is rapidly adopting advanced AI applications to deliver specialized, patient-centric care, demonstrating a commitment to global best practices and technological leadership. A notable example is The View Hospital, in affiliation with Cedars-Sinai, which performed Qatar’s first procedure using an AI-developed multifocal intraocular lens (IOL) (View, 2025).

Aman Hospital achieved a neurosurgical milestone by successfully performing cranial reconstruction using a customized 3D-printed PEEK (Polyetheretherketone) implant. This procedure is underpinned by an AI-driven pre-operative workflow, where machine learning algorithms are used to process complex patient CT/MRI scans, segment the anatomical structures, and generate the precise digital model required for the customized 3D print. This pioneering adoption of cutting-edge technology and superior biomaterials sets a new standard for advanced, high-precision surgical care within the nation’s private healthcare sector (Aman, 2025). Table 3 summarizes the status of major AI applications in Qatar.

**Table 3:** Comparative matrix of artificial intelligence (AI) applications in Qatar’s healthcare sector.

AI application	Clinical/operational domain	Status
Qatar Genome Program (QGP)	Genomics/data generation	Implemented
National EHR system	Data infrastructure	Implemented
AI for early detection of Type 1 diabetes (T1D)	Diagnostics/predictive health	Implemented/research
AI-enhanced diagnostic scoring (POST)	Personalized surgical planning/diagnostics	Implemented/research
AI-equipped advanced radiotherapy (Varian ethos)	Oncology/personalized treatment	Implemented
AI in drug design and diagnostics for colorectal cancer (CRC)	Research/drug development/diagnostics	Research/development
AI-developed multifocal intraocular lens (IOL)	Ophthalmology/personalized eye care	Implemented
Ai- driven chronic care digital twin (Lillia)	Chronic disease/management personalized care	Under development
System for integrated health analytics (SIHA)	Public health/decision support	Development/pilot
TASMU Smart Qatar (digital twin)	Predictive health/AI- assisted diagnosis	Research/development
AI-driven 3D surgical planning (implants)	Neurosurgery/surgical planning	Implemented/pilot
AI in robotics for surgical procedures	Surgical/operational domain	Widely adopted

This matrix provides a detailed overview of key AI projects, classifying them by application, their clinical or operational domain, and their current development status (e.g., implemented, research, under development/pilot).

## 5 Critical perspective on implementation barriers and regional context

### 5.1 Algorithmic bias and data standardization

The performance of AI models is strictly contingent upon the quality and type of their training data. In a genetically diverse area, the training of the AI models mainly on Western populations will lead to the introduction of bias that could be systemic (Cau et al., 2025). This will, in turn, affect the diagnosis and treatment of patients elsewhere in the Middle East, and specifically Qatar. The QGP, therefore, is not merely a research project but a foundational, risk-mitigating strategy to generate a representative national dataset that ensures the ethical and equitable performance of future AI applications in the country. The process of overcoming the challenges of interoperability and data standardization is now being dealt with more openly and directly. The healthcare system in Qatar is certainly advantageous as it is centralized with a national EHR system in place, however, the exchange of data between various platforms, institutions, and legacy systems remains a significant hurdle. The lack of standardized data formats and semantic interoperability can impede the development and deployment of scalable AI solutions, forcing developers to spend excessive time on data cleaning and harmonization rather than model development.

### 5.2 Data privacy and protection: a proactive framework

By building an enabling environment, the State of Qatar has paved the way for the country’s rapid technological growth. It was the first country in the Middle East to have enacted a data protection law, Personal Data Privacy Protection Law (PDPPL), in 2017 (CRA, 2025). The PDPPL provides that any collection and processing of personal data, including sensitive personal data concerning health, must be done in a lawful manner and with consent from the person whose data is collected and processed. This mature approach to governance provides exceptions to consent to data processing in certain cases, even including scientific research in the public interest, something that is particularly useful for large-scale projects like the Qatar Genome Program (QPHI, 2025). Such research, without this legal margin, would face immense practical and legal disadvantages. Hence, this fine balance demonstrates a mature stage in governance, trying to get regulation not to act as a hurdle to innovation but, on the contrary, as a facilitator carefully designed to mitigate risk and protect individual privacy (El-Khoury and Albarashdi, 2025). In addition to data privacy laws, Qatar has created a framework to address the ethical issues of AI and its safe use. National Cyber Security Agency (NCSA) issued the “Guidelines for Secure Usage and Adoption of AI,” which seeks to increase trust by encouraging the responsible use of AI and the mitigation of information security risks. These guidelines provide a set of core principles that include transparency, accountability, fairness, and robustness (NCGAA, 2025). The Qatar Ministry of Health in conjunction with the Communications Regulatory Authority (CRA) uses a risk-tiered system to regulate the use of AI in the healthcare field. The system ensures that AI of a higher level of risk, such as those designed for diagnostics and treatment, are subjected to more stringent evaluation as compared to those of lower risk, such as administrative aids (Solaiman et al., 2025).

### 5.3 Ethical considerations in a high-stakes domain

The National AI Strategy highlights the need for ethical, transparent and accountable AI systems that integrate the country’s culture and social norms. Universities along with other public sector institutions are taking part in the discussion (Qatar M., 2025; Solaiman et al., 2024). One of the centers involved in the dialogue is Hamad Bin Khalifa University (HBKU), which has organized a noteworthy conference on AI ethics to discuss the responsible development and deployment of technology taking into consideration value systems from different parts of the world (HBKU, 2025a). These criteria are part of the AI regulatory framework in Qatar alongside the national AI ethics charter. The demand for clarity, particularly in healthcare, is critical to achieving trust in AI systems. Its partners and the government accept the requirement that both patients and doctors be given explanations on AI models, their training data, and other relevant information to make sure those decisions are equitable and that lead to responsible outcomes (HBKU, 2025b).

### 5.4 Comparative regulatory benchmarks

To further strengthen Qatar’s emerging AI liability framework, a comparative analysis with the European Union’s (EU) recent legislative efforts provides a valuable benchmark, particularly for high-risk medical devices. The EU has established a dual regulatory approach: the EU AI Act and the revised Product Liability Directive (PLD). The AI Act classifies most AI systems used in medical devices as ‘high-risk,’ imposing strict requirements for data quality, transparency, and human oversight (Solaiman and Malik, 2025). Crucially, the revised PLD extends the principle of strict liability to defective software and AI systems, making it easier for patients to seek compensation for harm caused by an AI-driven medical device (Duffourc and Gerke, 2023; Nikolinakos, 2024). This dual regulatory approach provides a robust model for Qatar to consider in developing its own clear legal recourse, which is essential for fostering public trust in life critical AI applications (Vardas et al., 2025).

### 5.5 Comparative analysis of GCC AI healthcare strategies

Qatar’s model, may be contextualized through a comparative analysis of AI in healthcare strategies across the GCC, specifically focusing on the UAE and KSA. This comparison reveals that while all three nations are recognized as “Innovation Futurists” in healthcare due to their approaches to governance, regulation, and implementation diverge significantly (Solaiman et al., 2024). Qatar’s approach is characterized by a “top-down, centralized” model, as discussed earlier integrated projects like QGP, the national EHR system and integration tools (SIHA). This strategy prioritizes foundational data generation to ensure that AI models deployed in the country are trained on a representative population.

While, the UAE’s strategy, particularly in Dubai and Abu Dhabi, is characterized by a distributed governance model with Chief AI Officers embedded in every major ministry a pioneering approach initiated when the UAE appointed the world’s first Minister of Artificial Intelligence in 2017 (UAE, 2025). The Ministry of Health and Prevention (MOHAP) operates a dedicated AI Office that has deployed AI for communicable disease screening via X-ray analysis (MoHAP, 2020). and operates the “PaCE” (Smart Healthcare Operation Centre) to reduce emergency department waiting times (MoHAP, 2019).

Saudi Arabia, led by the Saudi Data & AI Authority (SDAIA), has adopted a highly centralized and comprehensive national data governance framework that positions data as a national asset (SDAIA, 2020). KSA healthcare AI strategy is exemplified by the King Faisal Specialist Hospital & Research Centre (KFSH&RC), which has developed over 30 AI-powered solutions in-house and operates a Centre for Healthcare Intelligence (CHI) with significant numbers of AI applications in medical imaging (KFSHRC, 2024a, 2024b, 2025).

These three distinct models Qatar’s data generation focus, Saudi Arabia’s operational efficiency emphasis, and the UAE’s innovation ecosystem approach represent different but complementary strategies within the GCC. The following Table 4 summarizes the key strategic differences.

**Table 4:** Key Strategic differences and complementarities in artificial intelligence (AI) healthcare strategies across the GCC.

Strategic dimension	Qatar	United Arab Emirates (UAE)	Saudi Arabia (KSA)
Governance model	Centralized, Top down (National AI strategy, QGP).	Decentralized/ Federated (Minister of State for AI, Regulatory Sandboxes).	Highly centralized (SDAIA, National data governance)
Data governance	First in the region to enact detailed data protection law; focus on generating representative national data (QGP).	Strong focus on cross border data transfers and attracting international data firms.	Comprehensive national data governance framework: data viewed as national asset.
Flagship focus	Precision Medicine (genomics/T1D), Specialized care (radiotherapy).	Public Health (communicable disease screening), Administrative Streamlining, Talent Attraction.	Administrative efficiency (patient flow), remote monitoring, robot assisted surgery.
Regulatory approach	Emerging, with focus on research guidelines and certification.	Regulatory sandboxes, rapid adoption of international standards.	Advanced guidance for AI-based medical devices (SFDA).

This table compares the approaches of Qatar, the UAE, and the KSA across four critical dimensions: governance model, data governance, flagship focus, and regulatory approach.

This analysis reveals that Qatar’s progress, while pioneering in its focus on genomic data and precision medicine, is part of a broader regional trend of GCC nations positioning themselves as global leaders in AI-driven healthcare. Qatar’s distinctive contribution lies in its integrated, national-scale approach to data generation and ownership, which addresses a critical gap in global AI development.

## 6 Catalyzing AI in Qatar’s healthcare sector: the human capital imperative

### 6.1 Addressing the knowledge deficit: AI perceptions among Qatar’s future healthcare workforce

The successful integration of AI technologies is fundamentally hindered by a significant human capital knowledge gap. This goes beyond simple upskilling and involves a deeper, more systemic issue of professional readiness. A core challenge is the dichotomy between clinical expertise and data science literacy. Clinicians, while possessing the domain knowledge essential for AI development and deployment, often lack the technical understanding to critically evaluate interpret, and trust AI outputs. Conversely, data scientists may not fully grasp the clinical context, leading to the development of models that are technically sound but not clinically relevant or safe. The nuanced impact is that this gap can lead to either an underutilization of AI tools due to clinician distrust or the misapplication of AI solutions that fail to address real-world patient needs. This human capital challenge is evident through recent studies that reveal a significant paradox within the emerging healthcare workforce: while there is a general awareness and receptiveness to AI, a substantial knowledge gap persists regarding its practical applications and conceptual underpinnings.

A foundational study by Ahmad et al. (2023) examined the perceptions of healthcare students including medical students at QU regarding the integration of AI into clinical practice. The primary purpose of this research was to assess students’ current knowledge of AI applications in medicine, while also identifying their attitudes and potential concerns, such as the impact on job security. While most students (92.6%) have heard of AI, only moderate proportions (39.5%) felt they had a good understanding of its concepts (Ahmad et al., 2023). The findings proposed to better prepare the future healthcare workforce for a technologically evolving environment, thereby aligning with Qatar’s broader national AI strategy (Ahmad et al., 2023). This knowledge deficit is not confined to medical students. A commentary, also by Ahmad et al. (2023) highlighted the necessity of preparing the next generation of nurses to effectively interact with and leverage AI. It highlighted a significant knowledge gap among nursing students and professionals concerning AI principles (Nashwan and Abujaber, 2025). Further evidence of this challenge is found in a pioneering cross-sectional study of dental students at Qatar University by Halat et al. (2024) that assessed dental students’ readiness, perceptions, and educational needs concerning AI in health education and practice (Halat et al., 2024). While dental students show an overall readiness for AI, they had less confidence in their practical skills and cognitive abilities with AI tools. The study emphasized an urgent need to incorporate AI into dental school curriculums, as over 80% of students wanted formal education to prepare them for AI’s role in clinical practice (Halat et al., 2024). Another study that involved more than 900 healthcare workers in Qatar found that there was a positive perception and moderate knowledge about AI among these workers. A large majority (72%) thought that AI would make healthcare better in the future, but 81.7% also said that AI had not been used in their practice yet. This huge difference between a positive attitude and limited practical use points out a major barrier to implementation: the non-existence of easy-to-use AI tools that are integrated into daily clinical workflows and the corresponding lack of CPD that is geared towards building skills and confidence (Al-Qudimat et al., 2025).

While Qatar’s National AI Strategy includes robust pillars for talent development, the unique demographic structure where non-citizens constitute the vast majority of the healthcare workforce presents a critical sustainability challenge that warrants explicit discussion (Rahman et al., 2023). The successful implementation and long-term maintenance of AI-driven services partially depend on the expertise of the workforce, including long-term residents and expatriate professionals (Albous et al., 2025). Beyond the scope of scholarship programs which will be addressed in a subsequent (section 6.4), Qatar’s strategic framework is bolstered by Law No. (10) of 2018, which permits the granting of Permanent Residency to scientists, academics, and healthcare professionals possessing specialized competencies (MOI, 2018). This legal pathway provides a critical mechanism for long-term talent retention, offering beneficiaries and their families stability through access to government-funded healthcare and education, as well as the right to invest in the national economy without a local partner (Beaugrand, 2023).

### 6.2 National initiatives for skill development

The human capital initiatives in Qatar are vital for meeting SDG 9’s goal of upgrading technological capabilities and increasing the number of research and development workers. By identifying and addressing the knowledge gap among future healthcare professionals, Qatar is systematically building a workforce that can effectively use and advance AI technologies. Foundational to this wide-scale national effort is the strategic and ongoing collaboration between the government and technology organizations, most notably in relation to its partnership with Microsoft. This partnership is designed to build digital skills and AI skills with special emphasis on enhancing the skills of senior leadership within the government sector so that these senior leaders will have the necessary skills to lead and oversee the implementation of large-scale digital transformation initiatives themselves, ensuring full top-down commitment and informed decision-making with respect to the adoption of AI (MCIT, 2025c).

Complementing these leadership-focused efforts, Qatar had launched an ambitious National Skilling Program to train 50,000 individuals in various digital skills by 2025. Within the ambit of this larger program is a sub-initiative to specialize in training 10,000 healthcare professionals in AI and telemedicine by the same year (MCIT, 2024; Microsoft, 2022).

This targeted approach underscores Qatar’s commitment to building a specialized healthcare workforce that is not only conversant with AI principles but also proficient in their practical application, thereby directly addressing the knowledge gaps and skill deficits highlighted in previous sections. While these initiatives represent a robust foundational commitment, future research could critically evaluate their pedagogical efficacy, scalability, and long-term impact on clinical outcomes and healthcare system efficiency using proposed key performance indicators as stated in Table 5.

**Table 5:** Proposed evaluation framework for tracking the success and impact of AI-enabled tools in healthcare.

Monitoring dimension	Measurable outcome	Rationale
Competency and proficiency	Post-training competency scores; Changes in self-reported confidence (e.g., pre- and post-intervention surveys).	To validate the effectiveness of educational content and delivery methods.
Adoption and utilization	Rate of adoption of AI-enabled tools in clinical practice; Changes in clinical workflow efficiency (e.g., time spent on planning, diagnosis).	To link human capital development to tangible operational and clinical improvements.
Clinical impact	Improvements in diagnostic accuracy or treatment planning quality (e.g., reduction in contouring time in radiotherapy).	To establish a direct correlation between workforce upskilling and enhanced patient outcomes.
Ethical and trust	Longitudinal surveys tracking clinician and patient trust in AI systems; Qualitative feedback on perceived ethical risks and liability concerns.	To ensure that AI integration is conducted responsibly and fosters a culture of trust and ethical use.

The framework is structured across four monitoring dimensions (competency, adoption, clinical impact, and ethical/trust), detailing the measurable outcomes and the rationale for their selection to ensure comprehensive assessment of AI implementation.

### 6.3 Continuous professional development (CPD): sustaining competence in a dynamic landscape

Due to the ever-increasing pace of AI technology evolution, CPD assumes extraordinary importance for maintaining and building AI skillsets for the present-day healthcare workforce. This pillar stresses lifelong opportunities for the professionals to keep up with new AI developments, address and refine respective skills, and realign clinical paradigms. The CPD efforts, offer varied modes such as online courses and workshops to pursue learning at their convenience, immersive learning globally recognized for hands-on training and simulations with AI tools, as well as collaborations amongst healthcare institutions, companies into tech, and academia (Charow et al., 2021). Such partnerships have proven to be effective for knowledge sharing, utilization of leading-edge AI platforms, and conduct of research on a collaborative basis. The hallmark of effective CPD, therefore, is that it is relevant to clinical practice, provides easy access, and generates knowledge and skills that are immediately put to use to advance patient care and enhance operational efficacy (Topol, 2019).

Qatar is addressing, in a proactive manner, the CPD needs in artificial intelligence so as to maintain alignment with the national vision and digital transformation agenda of the country. Taking into consideration of this AI dimension, it is seen that since AI would be a great agent in creating a diversified knowledge-based economy, multiple organizations are able to provide CPD programs that will ensure the workforce is enhanced for life and ready for the future of work. A prime instance of this is the offering of a workshop titled “Fundamentals of Artificial Intelligence in Healthcare with Practical Applications” at Weill Cornell Medicine-Qatar (WCM-Q) (WCM, 2025).

### 6.4 Incentives and career pathways: motivating engagement and retention

To equip a strong AI-enabled healthcare workforce, clear incentives and attractive career pathways must be put in place, which would encourage them to pursue AI and retain their workforce in the field. Following this, a number of universities and organizations in Qatar provide scholarships for degrees both at undergraduate and postgraduate levels in AI and its allied fields (CMUQ, 2025; UDST, 2025a). Few of these scholarships may include a service contract with a government entity after graduation to ensure that top talent is retained and contributes to Qatar’s development (MEHE, 2025; UDST, 2025b).

### 6.5 Addressing resistance and promoting adoption: fostering a culture of acceptance

This dilemma is not only in Qatar but a worldwide concern. AI’s resistance in healthcare is a global concern borne out of distrust and misunderstanding, on the sides of both medical practitioners and the public-at-large, and fueled by concerns over job security, mistakes, or errors, including the infamous “black box” problem (Khan et al., 2025). To curb these socio-cultural obstacles, the road map needs to create ethical governance, guarantee transparency with Explainable AI, and support a cooperative human-in-the-loop framework that positions AI to support rather than supplant clinicians and allied health professionals.

**Figure 2:**
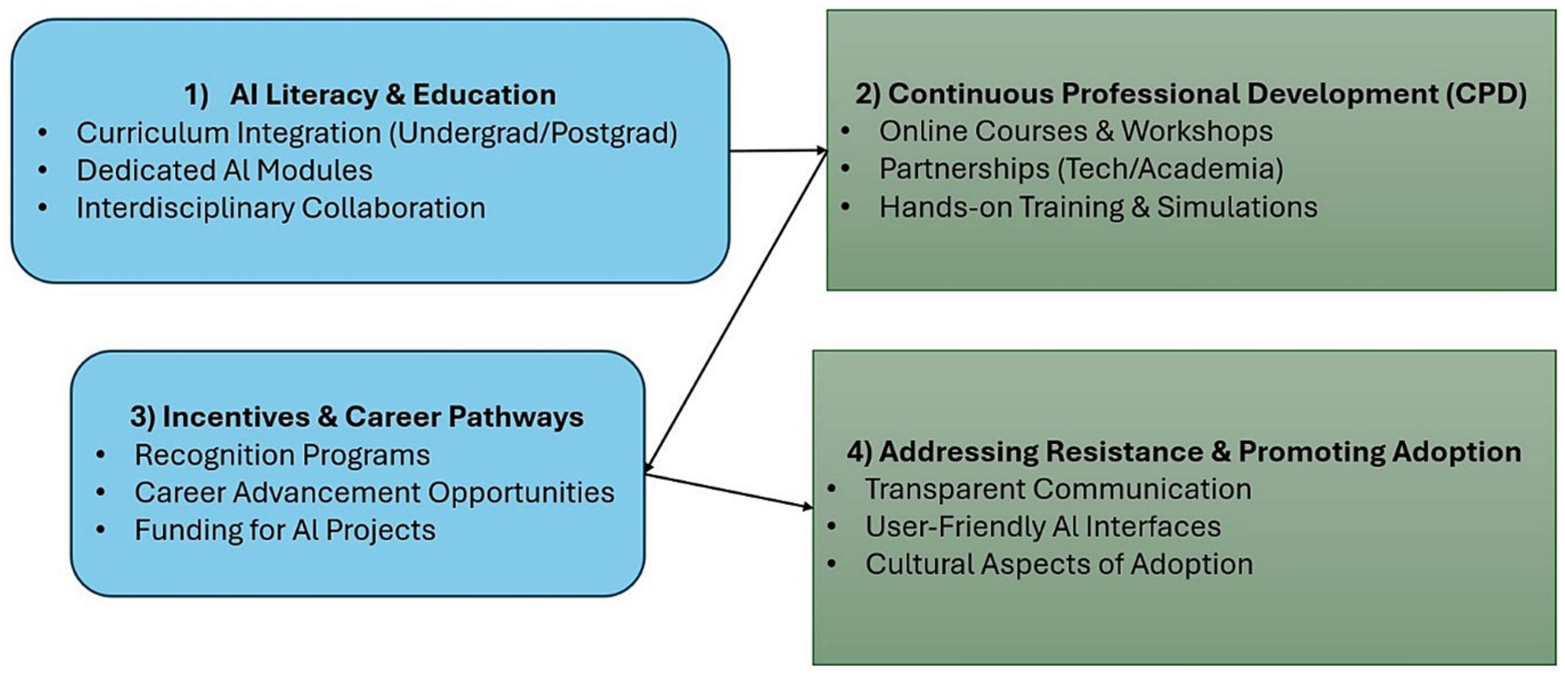
Human capital development framework.

The diagram (Figure 2) illustrates four interconnected pillars for fostering a culture of acceptance for AI in healthcare. These pillars are AI Literacy & Education: Focuses on curriculum integration, dedicated AI modules, and interdisciplinary collaboration. CPD: Highlights the importance of online courses, workshops, partnerships between academia and technology, and hands-on training. Incentives & Career Pathways: Suggests using recognition programs, creating career advancement opportunities, and funding AI projects to encourage adoption. Addressing Resistance & Promoting Adoption: Recommends using transparent communication, creating user-friendly AI interfaces, and addressing the cultural aspects of adoption.

### 6.6 Data and infrastructure

Qatar is actively investing in its technological base. A High-Performance Computing (HPC) Program aims to create a National HPC Program to bolster AI-optimized cloud capabilities. In addition, Qatar’s legal procedures are changing to support encryption and anonymization in place of compulsory local data storage, which speeds up international data collaboration (Hawkins, 2021; MCIT, 2025a, 2025b; Yuan, 2025). Beyond these foundational investments, the successful implementation of AI is critically dependent on a robust data governance framework. AI models are only as good as the data they are trained on. A lack of data quality, standardization, and interoperability create a flawed foundation for AI. The impact is that AI models trained on such data may produce biased or inaccurate predictions, ultimately jeopardizing patient safety. Without a cohesive data governance framework, the true value of AI in a decentralized healthcare system, with its siloed data, cannot be fully realized.

## 7 Conclusions and recommendations

**Figure 3:**
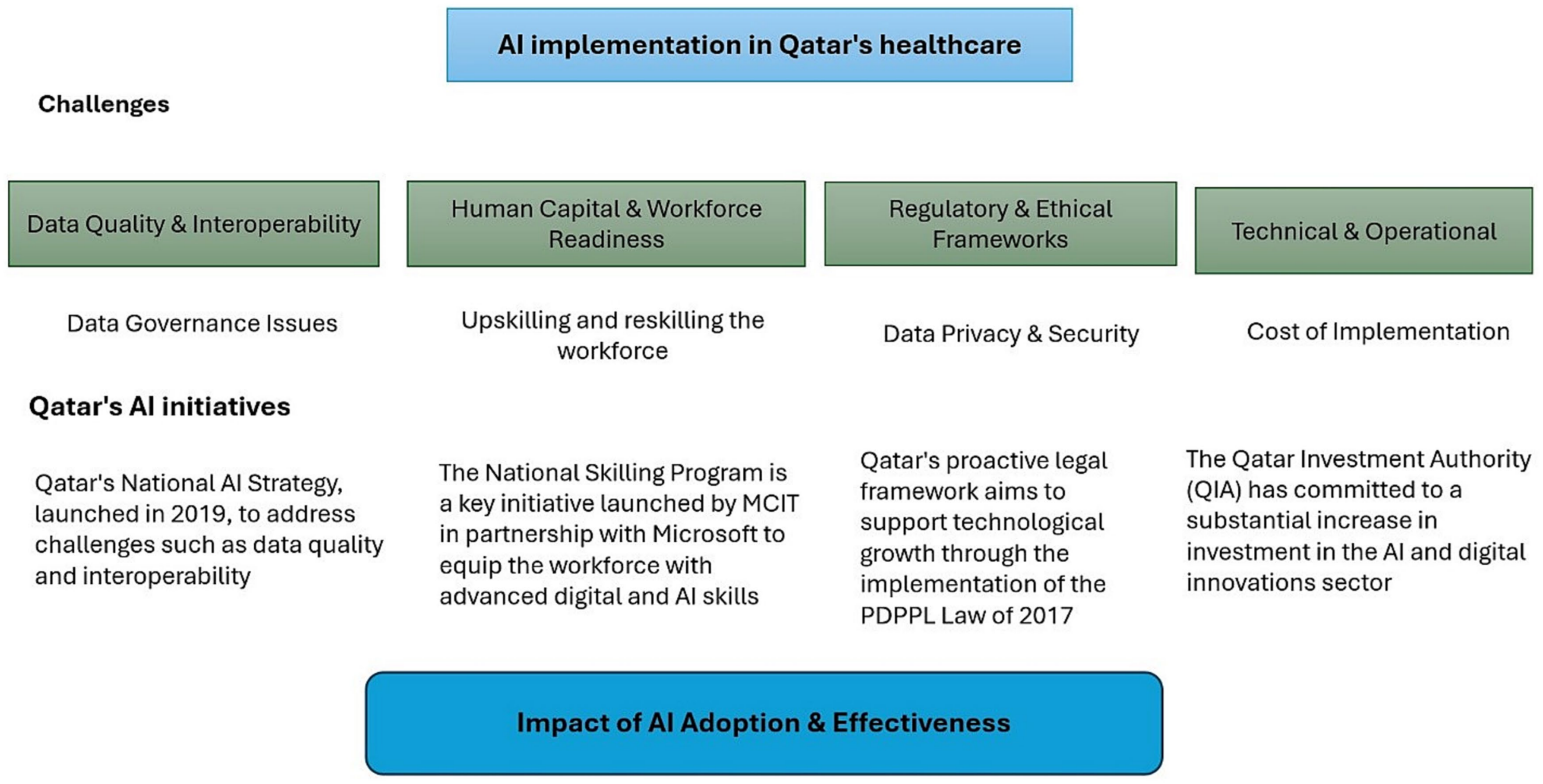
Challenges and initiatives in AI implementation in Qatar’s healthcare sector.

Qatar’s approach to leveraging AI in healthcare is a strategic, top-down effort, demonstrating clear foresight in aligning technology with national objectives (Figure 3).

With a strong digital infrastructure already in place a centrally developed EHR system has preempted problems like fragmented data and hence has carved the path for AI integration. Through the establishment of digital infrastructure, innovation in medicine, and a supportive regulatory environment, the country is transforming its health sector according to the global sustainable development vision. Challenges remain, but their very existence also offers a way forward for other countries wishing to work on technological frontiers toward a better future, since the linkages between these national level endeavors and international goals are so obvious. Lessons may be learned from the Qatar example on translating top-down initiatives into real-world programs, not only serving a local agenda but also a global one. While very successful, the strategic government-led initiatives have shed light on a major problem related to “human capital-based knowledge gaps.” Educational institutions need to take the lead in setting up AI curricula for the new-generation healthcare workforce to work alongside advanced technology. Ultimately, Qatar’s model serves as a pioneering case study, offering a blueprint for how a nation can effectively leverage centralized approach strategy and a forward-looking regulatory framework to not only adopt but to truly harness the transformative potential of artificial intelligence in healthcare.

### 7.1 Limitations of the study: the challenge of empirical validation

Though this paper provides a panoramic overview of Qatar’s strategic initiatives, a key limitation, which also reflects a broader challenge in the rapid deployment of AI technologies, is the inconsistent availability of published, peer-reviewed empirical evidence and quantitative outcome data for several highlighted AI applications. This limitation is not unique to Qatar only but reflects a broader challenge in the rapid deployment of AI technologies in healthcare sectors, as the case in many other high-income countries (HICs). A recent survey of US hospitals on the use and evaluation of predictive models. Reported concerns over ensuring AI models are Fair, Appropriate, Valid, Effective, and Safe (FAVES). The major limitation identified was the lack of local evaluation for bias and accuracy. Only 44% of hospitals using models reported local evaluation for bias. This lack of evaluation may lead to an organizational digital divide and the use of low-quality or biased models on different patient populations (Nong et al., 2025). Another study from East European Countries identifies barriers to using AI-based evidence for Health Technology Assessment (HTA). It classified barriers into five groups: data-related, methodological, technological, regulatory and policy-related, and human factor-related. Top barriers included lack of expertise and skills among decision-makers and practitioners (human factors), and issues with data reliability, validity, and fragmentation (data-related) (Tachkov et al., 2022). A scoping review examined the factors influencing the adoption of AI by clinicians in healthcare settings. It identified about 18 categories of barriers and facilitators including algorithmic bias and fairness, issues with transparency and explainability (the “black box” problem), interoperability with existing systems, and legal and ethical considerations (Hassan et al., 2024). Therefore, the distinction between the challenges faced by Qatar in deploying AI and those in other regions is best framed as one of degree, not of kind. The absence of such data makes it challenging to definitively assess the efficacy, cost-effectiveness, and scalability of all mentioned initiatives. Future research would significantly benefit from a concerted effort by Qatari institutions to publish empirical findings, including clinical trial results, real-world evidence, and detailed performance analyses of their AI applications. This would not only enhance academic rigor but also provide invaluable insights for other nations embarking on similar AI integration journeys in healthcare.

To sum up: The review calls for context-specific research on.

- Establishing a National AI Curriculum: To bridge the divide between theoretical understanding and practical implementation of AI within the healthcare sector.
- Establishing a National Digital Health Innovation Registry (DHIR): A limitation to national-scale strategic monitoring and coordination is the absence of a unified, mandatory AI initiative registry. A critical next step should be the establishment of a centralized national AI registration authority. This authority would be tasked with registering all new AI projects across every sector in Qatar, thereby streamlining oversight, ensuring adherence to national standards, minimizing duplication of efforts, and facilitating effective long-term follow up and impact assessment.
- Longitudinal studies: As a key metric for monitoring AI integration, within a context that frames Qatar’s overall approach as national model for global technological progress.

## Glossary

AI: Artificial Intelligence
SDG 9: UN Sustainable Development Goal 9
QNV-2030: Qatar National Vision 2030
NHS: National Health Strategy
NDS3: Third National Development Strategy
MOPH: Ministry of Public Health
EHR: Electronic Health Record
QGP: The Qatar Genome Project
MCIT: Ministry of Communications and Information Technology
QF: Qatar Foundation
QRDI: Qatar Research, Development and Innovation Council
QNRF: Qatar National Research Fund
ML: Machine Learning
T1D: Type 1 Diabetes
POST: Plate Objective Scoring Tool (terminology used in the context of pediatric urology)
CNN: Convolutional Neural Network
NCCCR: The National Center for Cancer Care and Research
HMC: Hamad Medical Corporation
OART: Online Adaptive Radiotherapy (terminology used in the context of Clinical Application Sites)
OARs: Organs at Risk (terminology used in the context of reducing radiation doses)
H&N: Head and Neck (terminology used in radiotherapy planning)
QU: Qatar University
CRC: Colorectal Cancer
IVF: *In Vitro* Fertilization
ICM: Inner Cell Mass (terminology used in embryo morphology assessment)
TE: Trophectoderm (terminology used in embryo morphology assessment)
ZP: Zona Pellucida (terminology used in embryo morphology assessment)
NICUs: Neonatal Intensive Care Units
TASMU: Smart Qatar
QCRI: Qatar Computing Research Institute
HBKU: Hamad Bin Khalifa University
PHCC: Primary Health Care Corporation
mHealth: Mobile Health
SIHA: System for Integrated Health Analytics
IOL: Intraocular Lens
PEEK: Polyetheretherketone (terminology used in Medical and Healthcare Applications)
PDPPL: Personal Data Privacy Protection Law
CRA: Communications Regulatory Authority
NCSA: National Cyber Security Agency
EU: European Union’s
PLD: Product Liability Directive
GCC: Gulf Cooperation Council
UAE: United Arab Emirates
KSA: Kingdom of Saudi Arabia
MOHAP: Ministry of Health and Prevention (UAE)
SDAIA: Saudi Data and Artificial Intelligence Authority
KFSH&RC: King Faisal Specialist Hospital & Research Centre
CHI: Centre for Healthcare Intelligence
SFDA: Saudi Food and Drug Authority
CPD: Continuous Professional Development
