# Supplementary material for: The strategic trajectory of artificial intelligence in Qatar’s healthcare sector: a model for UN Sustainable Development Goal 9
Source: Front Artif Intell. 2026 Jan 27;9:1702242. doi: 10.3389/frai.2026.1702242 (PMC12886428; doi:10.3389/frai.2026.1702242)
Supplement: Supplementary file 1 [file Data_Sheet_1.docx]

**Literature search and review strategy for the study**

A comprehensive search using multiple strategies was performed on various databases and platforms. This included PubMed, Scopus, IEEE Xplore, Google Scholar, and official websites of the government (e.g., Ministry of Public Health, Ministry of Communications and Information Technology, Sidra, Hamad Medical Corporation). In addition, official university websites (e.g., Qatar University, Hamad Bin Khalifa University, are also available).

- **Inclusion and Exclusion Criteria**

The search strategy employed Boolean operators (AND, OR) to link keywords across four core conceptual domains, ensuring a targeted and multidisciplinary retrieval of evidence:

1. Geographical & Contextual Focus: Terms such as 'Qatar' and 'UN Sustainable Development Goal 9 (SDG 9)' were used to anchor the findings in the national strategic and global development contexts.
2. Technological Intervention: This domain focused on the core subject matter, utilizing key terms like 'Artificial Intelligence (AI)', 'Digital Transformation', 'Precision Medicine', 'AI-based diagnostics', 'Machine Learning', 'genomics Qatar AI.' and 'Natural Language Processing (NLP)'.
3. Sectoral Focus: Specific healthcare applications and infrastructure were captured using terms such as 'Healthcare', 'clinical process developments', 'Electronic Health Record (EHR)', 'Qatar Genome Project (QGP)', 'Telemedicine', 'IVF', and 'AI in Diagnosis'.
4. Ethical & Governance Factors: To address the necessary supporting frameworks, this domain included terms like 'ethical and regulatory frameworks', 'privacy and security', 'algorithmic bias', and 'data governance'.
5. Synthesis of Empirical studies: To complement the review synthesis and provide a quantitative assessment of AI's clinical impact in Qatar, a systematic extraction of empirical data was performed on all included studies and reports that presented quantitative results. The objective was to identify, collate, and summarize key performance metrics of AI applications in clinical settings. Terms such as ' T1D ', ' 3D Printing ', ' T1D ' POST', ' ETHOSTM', ' CRC', 'TE', 'ICM' and ‘ZP' were selected based on highly restrictive processes.

In addition to academic sources, the search incorporated grey literature critical for understanding national policy and implementation. This included national strategy documents (QNV-2030, National AI Strategy, Digital Agenda 2030), reports from key national entities [ Hamad Medical Corporation (HMC), Sidra Medicine, Qatar Computing Research Institute (QCRI), and relevant legislative documents (Personal Data Privacy Protection Law (PDPPL)].

- **Exclusion Criteria**

1. Thematic Mismatch: articles that address AI or Digital Transformation, which topic is outside boarder the medical field, for instance cases of application of AI in finance, oil & gas or sport, will be excluded.
2. Non-Relevant Methodology: The documents will be non-relevant that are defined only by a quantitative systematic review or meta-analysis that is solely focused on clinical outcomes. This is because the final output is a policy-oriented narrative synthesis.
3. Redundant Content: Articles that are duplicates, written in non-English languages, or abstracts for which the full text is inaccessible will be excluded.

- **Data Extraction and Synthesis**

The literature selection process involved a multi-stage screening of titles/abstracts and full-text articles, prioritizing documents that strategically positioned AI and healthcare within the Qatari state under the broad framework of QNV-2030 and those detailing innovative clinical process developments or Implementation challenges relevant. This deliberate focus on strategic positioning and national implementation challenges even when specific quantitative empirical data was unavailable directly addressed the inherent challenge in this emerging field, where the rapid deployment of AI initiatives often outpaces the public dissemination of rigorous, peer-reviewed empirical data and quantitative clinical outcomes. Consequently, the final synthesis employed a narrative analysis approach, which is an intentional qualitative methodological choice to capture the nascent, top-down strategic landscape and regulatory foundations, thereby making a policy-focused narrative the most feasible form of initial analysis. The overarching goal was to provide a conceptual and contextual overview that identifies the nation's strategic trajectory, details the national model for sustainable development, and presents a comprehensive analysis of opportunities, challenges, and strategic recommendations, rather than executing a quantitative systematic review or meta-analysis.
